# The effects of spatial structure on a host-parasite model with horizontal and vertical transmission

**DOI:** 10.1007/s12080-026-00645-y

**Published:** 2026-10-10

**Authors:** Jack Woodruff, Alex Best

**Affiliations:** School of Mathematical and Physical Sciences, University of Sheffield, Sheffield, UK

**Keywords:** Vertical transmission, Spatial structure, Pair approximation, Horizontal transmission, Trade-off

## Abstract

Vertical transmission of an infectious disease from parent to offspring is a common transmission route in many systems. However, the majority of vertical transmission modelling has assumed a well-mixed population, and we do not know how the spatial structure present in most real-world populations will impact the dynamics of these systems. Here we investigate how spatial structure affects the dynamics of a pathogen with both horizontal and vertical transmission. We introduce a lattice model with a pair approximation that includes both local and global transmission and reproduction. We find that vertical transmission’s ability to determine pathogen invasion is dependant on spatial structure. When the majority of transmission and reproduction is local, vertical transmission can destabilise a host population to cause limit cycles. Given the advantages of a pathogen having both horizontal and vertical transmission routes, we extend the model to investigate the likelihood of a mutant strain with both transmission modes outcompeting a resident strain with only horizontal transmission. In the absence of cost the mutant always invades. When there is a trade-off between vertical and horizontal transmission, the mutant emerges when the cost to the horizontal transmission rate is not too large. Depending on how the mutant appears within the host population, it may have an initial advantage over the resident strain even if it cannot outcompete in the long-term. Our work demonstrates that the importance of vertical transmission within host-parasite dynamics is preserved when spatial structure is included.

**Supplementary Information:**

The online version contains supplementary material available at https://doi.org/10.1007/s12080-026-00645-y.

## Introduction

Vertical transmission occurs when a pathogen is transmitted from parent to offspring at or prior to birth (Burrell et al. 2017; Fine 1975, 1984). Vertical transmission can be termed perfect if all offspring of an infected host are born infected, and imperfect if only a proportion of the offspring are born infected. For plant pathogens a vertically-transmitted disease is spread via seeds, and can cause outbreaks of disease (Dombrovsky and Smith 2017; Maule and Wang 1996; Pagán 2022). For animals, depending on the reproductive processes, the virus may be passed to offspring during fetal development, egg development, birth or postnatally, and the virus can come from either parent (Chitnis et al. 2013; Hanson et al. 2016; Lamond and Freitag 2018; Mims 1981). Notable human diseases that are vertically transmitted (Parums 2024) include HIV/AIDS (Cardenas et al. 2023) and Hepatitis B (Riches et al. 2025). The other main transmission mode is horizontal transmission, when an infected host passes disease to a susceptible host already in the population, such as via aerosol, water or sexual contact (Antonovics et al. 2017; Fine 1975). It is common for a pathogen to have a mixture of both transmission modes, even if transmission via one mode is relatively rare compared to the other (Ebert 2013). Theory shows that vertical transmission allows pathogens to persist in a wider range of ecological conditions (Altizer and Augustine 1997).

Mathematical models of vertical transmission are somewhat under-represented compared to horizontal transmission models. Where they do exist, analysis of mean-field models with vertical transmission have given insights into the influence of this transmission mode on population dynamics (Altizer and Augustine 1997; Busenberg et al. 1983; Gao and Hethcote 1992; Li et al. 2001; Lipsitch et al. 1995), as well as provided insights into specific diseases including Dengue (Adams and Boots 2010; Ahman et al. 2025; Zou et al. 2018), Zika (Agusto et al. 2017; Wiratsudakul et al. 2018), HIV/AIDS (Naresh et al. 2006; Rasheed et al. 2025) and Hepatitis B (Anley et al. 2023; Pang et al. 2010). In general, if a pathogen reduces the net growth of the host, it cannot invade via vertical transmission alone; horizontal transmission is required (Busenberg and Cooke 1993; Lipsitch et al. 1995). In contrast, a pathogen with only horizontal transmission is able to invade (Kermack and Mckendrick 1927), but when vertical transmission is present it can be a determinant in pathogen invasion (Altizer and Augustine 1997; Busenberg et al. 1983). Assuming both modes are present, the two transmission routes can have different roles in the course of a disease outbreak. While horizontal transmission may be critical during the early stages of an outbreak, vertical transmission can have a more significant role once the disease is established (Lipsitch et al. 1995). Vertical transmission can also allow the pathogen to persist at lower levels of host density (Anderson and May 1981) and a wider range of ecological conditions (Altizer and Augustine 1997). In the case of perfect vertical transmission and no recovery, an outbreak could lead to total infection of a population (Lipsitch et al. 1995). For pathogens with both horizontal and vertical transmission routes, theoretical and experimental studies have considered the possibility of a trade-off between the two transmission modes (García-Ordóñez and Pagán 2024; Magalon et al. 2010; Stewart et al. 2005; Turner et al. 1998; Zilio et al. 2018). In a mean-field model with such a trade-off, bistability has been found, whereby depending on the environment, a pathogen can evolve high horizontal transmission and virulence with low vertical transmission or low horizontal transmission and virulence with high vertical transmission (Bosch et al. 2010).

The majority of ecological interactions are subject to spatial structure, particularly the horizontal transmission of disease, which often occurs through direct contact between susceptible and infected hosts. Spatial structure is particularly important in systems where individual hosts are static, such as plants. Furthermore, models that account for spatial structure behave differently to their mean-field counterparts. Differing behaviours may include the occurrence of limit cycles and parasite-driven extinction (Best et al. 2012; Webb et al. 2007a, b). Spatial structure can be introduced into models through a variety of methods, one of which is the lattice approach with a pair approximation (Filipe and Gibson 1998; Matsuda et al. 1992; Satō et al. 1994). Using this approach, recent research has investigated how spatial structure affects host-parasite dynamics with only horizontal transmission by varying the proportion of local and global reproduction and transmission (Maltz and Fabricius 2016; Webb et al. 2007a; Wren and Best 2021). These studies have found that the proportion of local horizontal transmission has a larger impact than the proportion of local reproduction on the invasion boundary (Webb et al. 2007a). In contrast, the proportion of local reproduction has a larger effect on the occurrence of endemic limit cycles (Webb et al. 2007a).

While most theoretical studies of vertical transmission use a mean-field model, some have included spatial structure (Schinazi 2000; Silva et al. 2017; Su et al. 2019). In one such study of a lattice-based infectious disease model with perfect vertical transmission and no recovery, four steady states were identified (extinction, disease-free, endemic and total infection) and limit cycles were not found (Silva et al. 2017). Given that the birth rate is sufficient enough that extinction does not occur, the fecundity of infected hosts and the horizontal transmission rate are key parameters in determining disease invasion and whether total infection occurs (Su et al. 2019). For a stochastic model on a one-dimensional lattice where vertical transmission is imperfect and there is no recovery, it has been shown that if vertical transmission is below a threshold, then even for high horizontal transmission the disease will not persist (Schinazi 2000). When there is habitat loss, vertical transmission can play a more important role in whether the pathogen invades, due to the potential unavailability of susceptible hosts (Su et al. 2019). Despite these studies, a lattice-based deterministic model that allows for imperfect vertical transmission has not yet been considered, and it is unknown how imperfect vertical transmission will affect the behaviour of disease dynamics in spatially-structured populations.

Here we introduce such a model that also allows for both local and global horizontal transmission and reproduction. In Section “Model”, the model is introduced. Section “Results” presents the key results from our analysis, including an extension of the model that allows for the emergence of a mutant with vertical transmission in a population where it is otherwise absent. Section “Discussion” discusses the conclusions.

## Model

### Mean-field model

Consider a population comprised of susceptible (*S*) and infected (*I*) hosts, with a total population size *N*. For clarity, initially we assume there are no local interactions. The model is given by the following ordinary differential equations where dots represent the derivative with respect to time,

1 $$\begin{aligned} \dot{S}&= b(1-N/K)(S + f(1-\rho )I) - \beta SI - dS + \gamma I \end{aligned}$$

2 $$\begin{aligned} \dot{I}&= bf(1-N/K)\rho I + \beta SI - (d+\alpha )I - \gamma I . \end{aligned}$$

We assume horizontal transmission is density-dependant with co-efficient *β * and recovery rate *γ *. Reproduction is limited by carrying capacity *K* and occurs at rate *b* for susceptibles and *bf* for infecteds where *f∈ [0,1]* is fecundity. The probability of vertical transmission occurring is *ρ *. Natural death occurs at rate *d* with virulence, *α *, for infected hosts. Lipsitch et al. (1995) provides a detailed analysis of the dynamics of Eqs. 1-2. Briefly, there are three steady states of this model, extinction, disease-free and endemic. If vertical transmission is perfect, total infection is possible. Limit cycles and parasite-driven extinction do not occur.

### Pair-approximation model

We now introduce the spatial structure. Consider a square lattice, with periodic boundary conditions, where each site is empty (0) or occupied by a susceptible (*S*) or infected (*I*) host (Matsuda et al. 1992; Satō et al. 1994). Interactions are local if they occur between a site and one of its four nearest-neighbours, whereas interactions are global if they occur between two random sites. Both reproduction and horizontal transmission may occur locally or globally. The proportion of reproduction that occurs locally is $L_R$, whereby offspring occupy a neighbouring empty site. For example, for local birth, an (*S*) site reproduces into a neighbouring (0) site, such that an (*S*0) pair becomes an (*SS*) pair. Similarly $L_T$ is the proportion of horizontal transmission that occurs between an infected and a susceptible neighbour (i.e. an (*SI*) pair becomes an (*II*) pair). Note that $L_R = L_T = 0$ is equivalent to the mean-field case. Therefore, local reproduction of susceptible and infected hosts occurs at rates $bL_R$ and $bf L_R$ respectively, where $0\le L_R \le 1$, and hosts reproduce into random empty sites at rates $b(1-L_R)$ and $bf(1-L_R)$ respectively. Vertical transmission occurs locally at rate $\rho bf L_R$ and globally at rate $\rho b f(1-L_R)$, otherwise infected offspring are born susceptible. As in Eqs. 1-2, we assume horizontal transmission is density-dependent with it occurring locally between infected and susceptible neighbours at rate $\beta L_T$ and globally at rate $\beta (1-L_T)$ for $0\le L_T \le 1$. All other parameters are the same as for Eqs. 1-2 and are defined in Table 1.

**Table 1:** The parameters for the spatially-structured model

Parameter	Definition
*b*	Birth rate
*f*	Fecundity
*d*	Death rate
*α *	Virulence
*γ *	Recovery rate
*β *	Horizontal transmission rate
*ρ *	Probability of vertical transmission
$L_R$	Probability of local reproduction
$L_T$	Probability of local transmission

The probability a site is in state (*i*) is defined as $P_i$ for $i \in \{0,S,I\}$. Similarly, the probability a neighbouring pair of sites are in states (*ij*) is $P_{ij}$ where $i,j \in \{0,S,I\}$. For each pair of sites in state (*ij*), $q_{i/j}$ is the conditional probability that for a randomly chosen site in state (*j*), a randomly chosen neighbour is in state (*i*). Note that $q_{i/j} = P_{ij}/P_j$. Similarly, $q_{k/ij}$ is the conditional probability that given an (*ij*) pair, a randomly chosen neighbour of the site in state (*i*) is in state (*k*). Using these definitions, we define a system with both local and global reproduction and horizontal transmission, using the following ordinary differential equations,

3 $$\begin{aligned} \dot{P_0}&= (d+\alpha )P_I+dP_S - b\big [L_R(q_{S/0}+ f q_{I/0}) + (1-L_R)( P_S+ f P_I)\big ]P_0 \end{aligned}$$

4 $$\begin{aligned} \dot{P_S}&= b \big [ L_R(q_{S/0}+ f(1-\rho ) q_{I/0}) +(1-L_R)(P_S+f(1-\rho )P_I ) \big ]P_0 \\ &\quad \quad - \beta \big [ L_T q_{S/I}+(1-L_T)P_S\big ]P_I-d P_S +\gamma P_I \end{aligned}$$

5 $$\begin{aligned} \dot{P_I}&= bf \rho \big [L_R q_{I/0}+(1-L_R)P_I\big ]P_0+ \beta \big [L_T q_{S/I} +(1-L_T)P_S\big ] P_I \\&\quad \quad -(d+\alpha + \gamma )P_I . \nonumber \end{aligned}$$

This system is not closed because Eqs. 3-5 depend upon the pair densities, due to the conditional probabilities. For example $q_{S/I} = P_{SI}/P_I$. The dynamics of the pair probabilities are defined as,

6 $$\begin{aligned} \dot{P_{00}}&= 2 (d+\alpha ) P_{I0} +2 d P_{S0} -2 b \big [ L_R (3/4) ( q_{S/00} + f q_{I/00}) \nonumber \\&\quad \quad + (1-L_R) ( P_S + f P_I) \big ] P_{00} \end{aligned}$$

7 $$\begin{aligned} \dot{P_{S0}}&= b \big [ L_R (3/4) ( q_{S/00} + f (1-\rho ) q_{I/00}) +(1-L_R) ( P_S +f (1-\rho ) P_I) \big ] P_{00} \nonumber \\&\quad \quad + \gamma P_{I0} +d P_{SS} +(d+\alpha ) P_{SI}- d P_{S0} - \beta \big [ L_T (3/4) q_{I/S0} \\ &\quad \quad +(1-L_T) P_I \big ] P_{S0} - b \big [L_R (( 1/4 + (3/4)q_{S/0S}) \nonumber \\&\quad \quad +f (3/4) q_{I/0S}) +(1-L_R) (P_S+ f P_I)\big ] P_{S0}\nonumber \end{aligned}$$

8 $$\begin{aligned} \dot{P_{I0}}&= bf\rho \big [L_R (3/4) q_{I/00} +(1-L_R)P_I \big ] P_{00}+ \beta \big [ L_T (3/4) q_{I/S0} \nonumber \\&\quad \quad +(1-L_T)P_I \big ] P_{S0}+d P_{SI}+(d+\alpha ) P_{II}-(d+\alpha +\gamma ) P_{I0} \nonumber \\&\quad \quad - b \big [ L_R (f(1/4 + (3/4) q_{I/0I})+(3/4) q_{S/0I})+(1-L_R)(P_S+ f P_I)\big ] P_{I0} \end{aligned}$$

9 $$\begin{aligned} \dot{P_{SI}}&= b \big [L_R ((3/4) q_{S/0I}+f(1-\rho ) (1/4 + (3/4) q_{I/0I}))+(1-L_R) (P_S \nonumber \\&\quad \quad +f(1-\rho ) P_I)\big ] P_{I0} +bf \rho \big [L_R (3/4) q_{I/0S}+(1-L_R)P_I \big ] P_{S0} \\ &\quad \quad +\beta \big [ L_T (3/4) q_{I/SS}+(1-L_T) P_I \big ] P_{SS} + \gamma P_{II}-(2 d+\alpha + \gamma ) P_{SI}\nonumber \\&\quad \quad - \beta \big [ L_T (1/4 + (3/4) q_{I/SI}) +(1-L_T) P_I \big ]P_{SI}\nonumber \\ \end{aligned}$$

10 $$\begin{aligned}\dot{P_{SS}}&= 2 b \big [L_R (1/4 +(3/4) q_{S/0S} +f(3/4) (1-\rho ) q_{I/0S}) + (1-L_R) ( P_S\nonumber \\&\quad \quad +f(1-\rho ) P_I)\big ] P_{S0}+2 \gamma P_{SI}-2 d P_{SS}-2 \beta \big [L_T (3/4) q_{I/SS} \\ \end{aligned}$$

11 $$\begin{aligned} \dot{P_{II}}&= 2 bf \rho \big [L_R (1/4 + (3/4) q_{I/0I}) + (1-L_R)P_I \big ] P_{I0}\nonumber \\&\quad \quad +2 \beta \big [L_T (1/4 +(3/4) q_{I/SI})+(1-L_T)P_I \big ] P_{SI}-2 (d+\alpha + \gamma ) P_{II} . \end{aligned}$$

We also have that,

12 $$\begin{aligned}&\dot{P}_{S0} = \dot{P}_{0S}, \quad \dot{P}_{I0} = \dot{P}_{0I}, \quad \dot{P}_{IS} =\dot{P}_{SI}, \end{aligned}$$

13 $$\begin{aligned}&P_0 + P_S + P_I = 1, \end{aligned}$$

14 $$\begin{aligned}&P_{00}+P_{SS}+P_{II}+2(P_{I0}+P_{S0}+P_{SI}) = 1. \end{aligned}$$

Within the conditional probabilities in Eqs. 6-11 are the triplet densities. For example, $q_{I/SI} = P_{ISI}/P_{SI}$. To close the system we use a pair approximation (Matsuda et al. 1992; Satō et al. 1994), such that,

$$\begin{aligned} q_{k/ij}=q_{k/i} \quad \forall \quad i,j,k \in \{0,S,I\}. \end{aligned}$$

This follows the intuition that the states of the sites in state (*i*) and state (*k*) is of greater importance than the state of the more distant site in state in (*j*). The validity of this approximation is shown by comparing the numerically simulated deterministic model to a spatially explicit stochastic simulation. The stochastic simulation is constructed using the Gillespie Algorithm (Gillespie 1977), with *τ *-leaping to increase the speed of each simulation run (Gillespie 2001).

## Results

### Invasion and equilibria

**Fig. 1:**
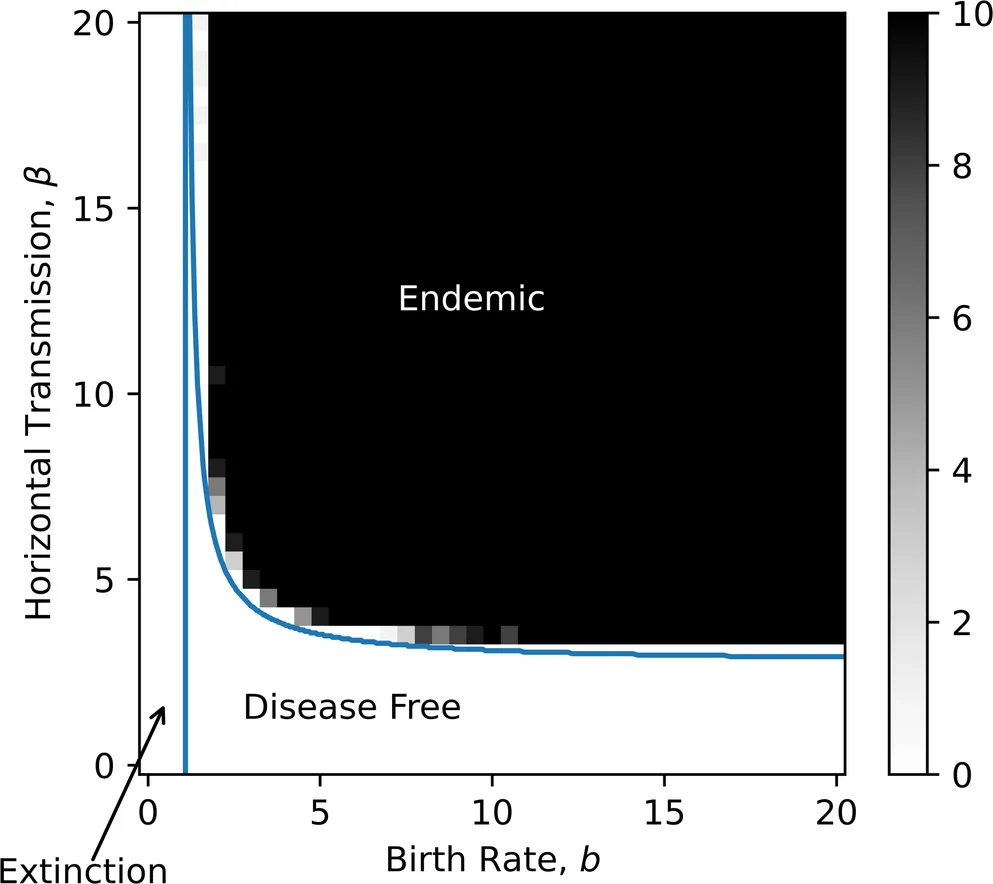
Equilibrium classification diagram in the *b*-*β * parameter space. The solid lines represent the invasion and extinction thresholds derived from numerical simulations of the deterministic model. The black and white shading shows the number of ten stochastic runs that resulted in pathogen invasion. The fixed parameters are $f=0.5, d=1, \alpha = 0.5, \gamma = 1.5, \rho = 0.75, L_R = L_T = 0.5$. For the deterministic model the initial conditions are $(P_0,P_S,P_I) = (0.1,0.8,0.1)$ with the initial pair probabilities as $P_{ij} = P_i P_j$ for $i,j \in \{0,S,I\}$

For the fixed parameters in Fig. 1, there are three possible outcomes of the model: extinction, disease-free and endemic. The extinction boundary was found by determining the stability of the Jacobian. The invasion boundary was verified using steady state values from numerical simulations. For this parameter set, initial conditions did not affect the steady state value of the model. In each case a unique equilibrium was observed once the simulation had run for a sufficiently large number of time units. Figure 1 shows the invasion boundary predicted by the deterministic model is close to that of the stochastic model. Limit cycles do not occur for the parameter values used here, but are found for alternative parameter regions considered later in this section. Parasite-driven extinction has been observed within spatial models with density-dependent transmission. However, total castration or extremely low levels of infected reproduction are required for this steady states existence (Webb et al. 2007b). To observe the effects of vertical transmission, a sufficient level of infected reproduction is required, thereby eliminating the possibility of this steady state within our analysis.

**Fig. 2:**
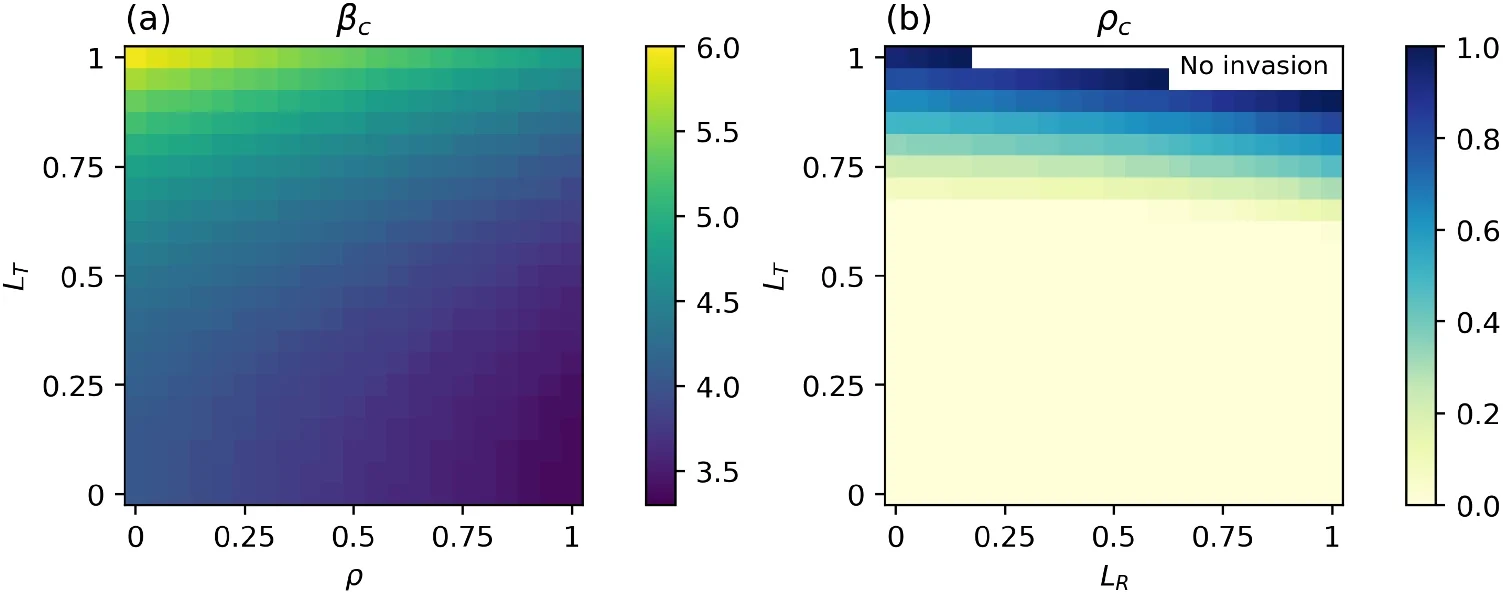
**a** Heatmap showing how $L_T$ and *ρ * affect the critical horizontal transmission rate, $\beta _c$. **b** Heatmap showing how $L_R$ and $L_T$ affect the critical probability of vertical transmission, $\rho _c$. The fixed parameters are $b = 4, f = 0.5, d = 1, \alpha = 0.5, \gamma = 1.5$ and (a) $L_R = 0.5$ (b) *β = 4.6* with initial conditions as in Fig. 1

The probability of vertical transmission can determine whether the pathogen invades. The minimum value of *ρ * for which this occurs is the critical probability of vertical transmission, $\rho _c$. The critical horizontal transmission rate, $\beta _c$, is defined in a similar way. Focussing close to the invasion boundary, Fig 2a shows that increasing *ρ * decreases $\beta _c$ for all values of $L_T$. Figure 2b demonstrates that in more localised systems $\rho _c$ is higher, with $L_T$ having a greater effect compared to $L_R$. For this parameter set, invasion does not occur in the most localised system and vertical transmission is not required when horizontal transmission is predominantly global.

**Fig. 3:**
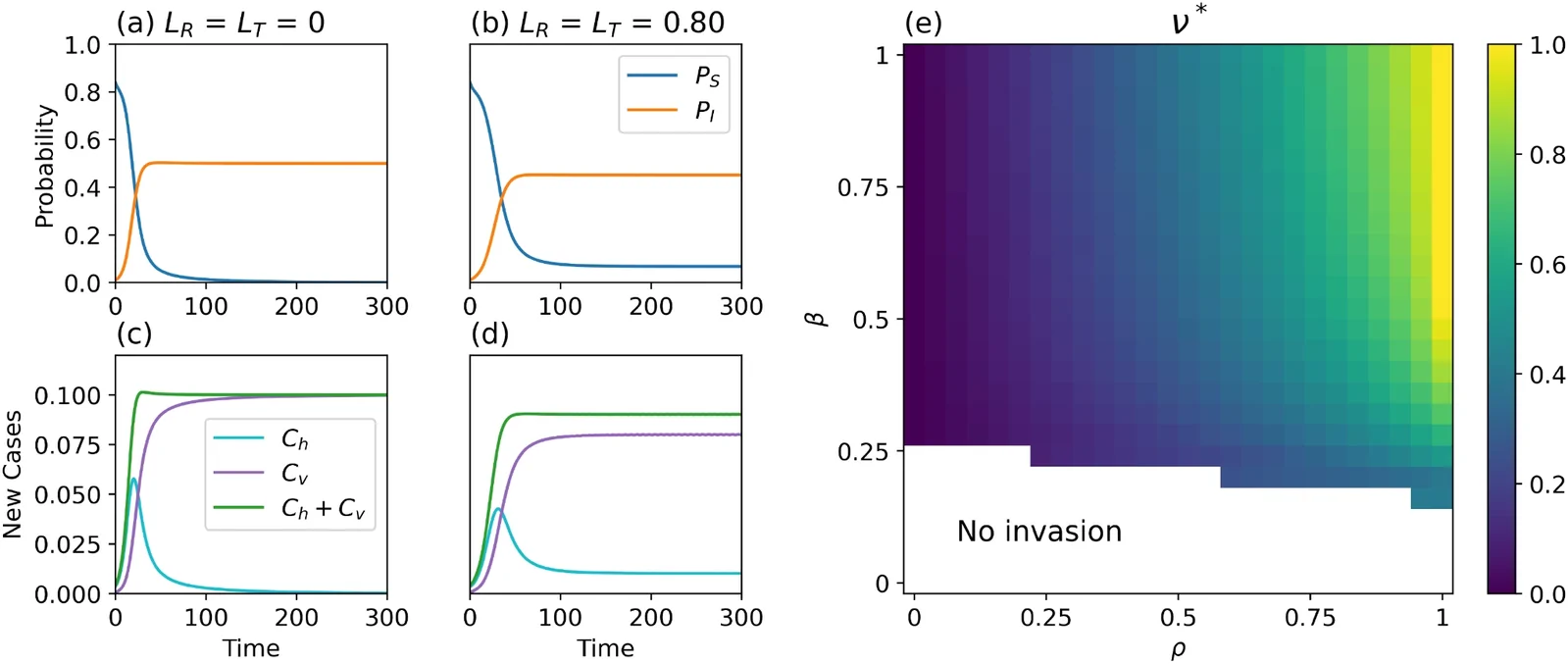
**a** and **b** show the time evolutions of the model with **c** and **d** showing the corresponding new cases over time respectively. **e** shows how the proportion of vertical transmission at the steady state, $\nu ^*$, varies in the *ρ *-*β * parameter space. The fixed parameter values are taken from Lipsitch et al. (1995) Fig. 1 with $f = 2/3, d=0.1, \alpha = 0.1, \gamma = 0, \rho = 1$ with (a-d) *b=0.6* and *β = 0.42*. The spatial parameters are (a, c) $L_T = L_R = 0$ and (b, d, e) $L_R = L_T = 0.8$. For both cases the initial conditions are $(P_0,P_S,P_I) = (0.15,0.84,0.01)$ with the initial pair probabilities as $P_{ij} = P_i P_j$ for $i,j \in \{0,S,I\}$

To compare the horizontal and vertical transmission routes, we look at the number of new cases over time, $C_h(t)$ and $C_v(t)$, for horizontal and vertical transmission respectively. Similarly to Lipsitch et al. (1995), these are defined as,

15 $$\begin{aligned} C_h(t)&= \beta [L_T q_{S/I} + (1-L_T)P_S]P_I \end{aligned}$$

16 $$\begin{aligned} C_v(t)&= bf\rho [L_R q_{I/0} + (1-L_R)P_I]P_0 \end{aligned}$$

Previous work has shown that in mean-field models with horizontal and vertical transmission, there are parameter values that result in only vertical transmission contributing to new infections at the steady state (Lipsitch et al. 1995). One such set is shown in Fig. 3a and c. However, in the equivalent spatial model this does not happen. As shown in Fig. 3d, horizontal transmission contributes new cases at the steady state. In the spatially-structured model there are a higher number of susceptible hosts at the endemic fixed point, thus increasing $C_h$ and lowering $C_v$.

Consider a time point, $t_1$, that is sufficiently large so that the system has reached a steady state. The proportion of vertical transmission at the steady state is given by

17 $$\begin{aligned} \nu ^* = \frac{C_v(t_1)}{C_v(t_1)+C_h(t_1)}. \end{aligned}$$

Figure 3e shows that, as expected, increasing *ρ * results in more vertical transmission at the steady state. Perhaps unexpectedly, increasing *β * also causes $\nu ^*$ to increase. Since increasing *β * reduces the susceptible population at the steady state, $C_h$ is also lower at the steady state, and therefore $\nu ^*$ increases. Figure 3d shows that initially $C_h$ is higher than $C_v$, suggesting that the invasion of the pathogen depends more on new cases arising via horizontal transmission. This explains why in Fig. 2b we see $L_T$ having a larger affect on $\rho _c$ compared to $L_R$, even though $L_R$ is more closely related to *ρ *.

**Fig. 4:**
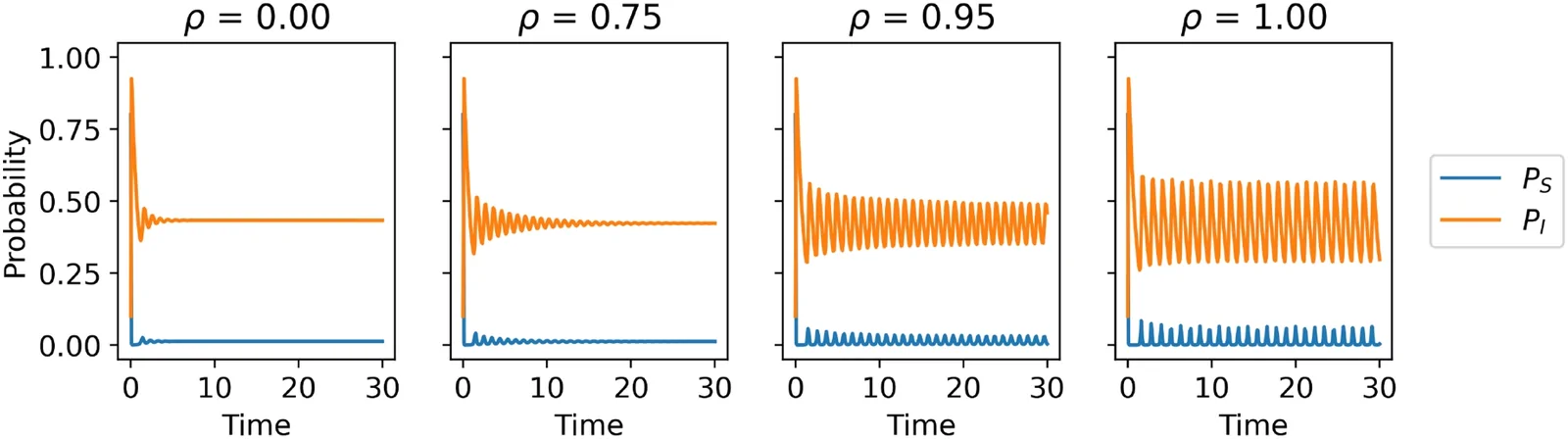
Time evolutions of the model for cases when dampened oscillations or limit cycles occur. The fixed parameters are $b = 100, f = 0.001, d= 1, \alpha = 0.05, \gamma = 0.0005, \beta = 100, L_R = L_T = 0.95$ with $\rho = \{0,0.75,0.95,1\}$. The initial conditions are the same as Fig. 1

As well as the steady states shown in Fig. 1, the model also gives rise to limit cycles. To find the amplitude of the limit cycles, we ran the numerical simulation of the model (Eqs. 3-11) until *t=2000*. Over the final 100 time units, we found the maximum and minimum values of $P_I$ and divided by 2. We found that cycles occur when *f, α * and *γ * are very small and *b* and *β * large. Figure 4 further demonstrates that limit cycles are more likely when *ρ * is high. When cycles occur, decreasing *ρ * decreases the amplitude of cycles, as shown in Fig. 4. This suggests that susceptible offspring from infected hosts can stabilise cycles.

**Fig. 5:**
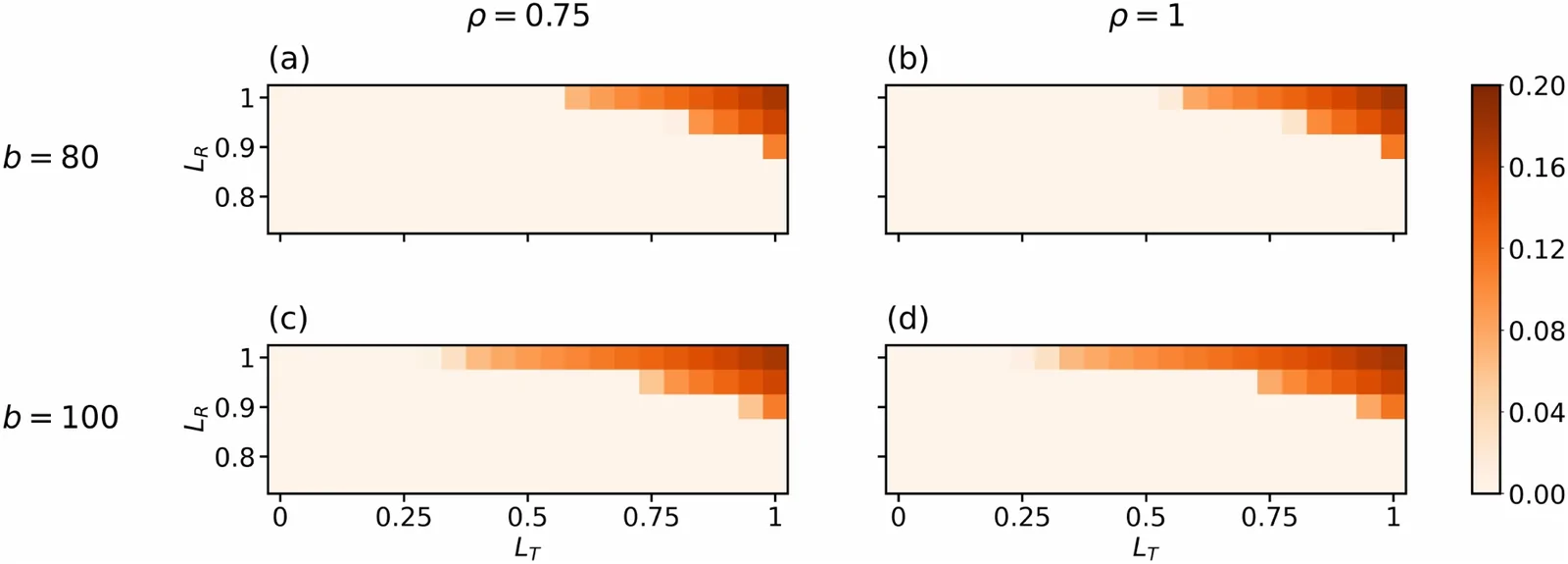
Heatmaps showing the amplitude of limit cycles. The fixed parameters are $f = 1\times 10^-5, d = 1, \alpha = 0.05, \gamma = 0.0005$ and *β = 100*. The initial conditions are the same as Fig. 1

Figure 5 shows that $L_R$ and $L_T$ need to be high for limit cycles to occur, with a larger amplitude in more localised systems. $L_R$ has a larger influence on whether limit cycles occur compared to $L_T$. In Fig. 5, it is required that $L_R \ge 0.9$. When reproduction is entirely local ($L_R=1$), then limit cycles can continue to occur even when horizontal transmission is mainly global ($L_T < 0.5$). However, once there is even a small amount of global reproduction, limit cycles only occur when the majority of transmission is local. In Appendix A, we further investigate how other model parameters affect the occurrence and amplitude of limit cycles. We find $\rho , f, L_R$ and $L_T$ to be particularly important in determining whether limit cycles occur.

### Invasion of a vertically transmitted strain

We now ask how easily a strategy of vertical transmission can emerge in a pathogen population that previously only had horizontal transmission. As an initial investigation, we examine the conditions required for a mutant with vertical and horizontal transmission to displace a strain with only horizontal transmission. Consider a resident strain that is only transmitted horizontally, where $P_I$ is the probability a site is occupied by a host infected with the resident strain. $P_V$ is the probability a site is occupied by a host infected with the mutant strain that has vertical transmission as an additional transmission mode. $P_S$ and $P_0$ are as defined in Section “Model”. The pair probabilities are $P_{ij}$ for $i,j \in \{0,S,I,V\}$. The system is defined by the following ordinary differential equations,

18 $$\begin{aligned} \dot{P_0}&= dP_S + (d+\alpha _I)P_I + (d+\alpha _V)P_V - b(q_{S/0} + fq_{I/0} + fq_{V/0})P_0 \end{aligned}$$

19 $$\begin{aligned} \dot{P_S}&= b (q_{S/0} +fq_{I/0} +f(1-\rho )q_{V/0})P_0 + \gamma _I P_I + \gamma _V P_V - dP_S \\ &\quad \quad -\beta _I q_{S/I} P_I - \beta _V q_{S/V} P_V \nonumber \end{aligned}$$

20 $$\begin{aligned} \dot{P_I}&= \beta _I q_{S/I} P_I - (d+\alpha _I+\gamma _I)P_I\end{aligned}$$

21 $$\begin{aligned} \dot{P_V}&= b f \rho q_{V/0} P_0 + \beta _V q_{S/V} P_V - (d+\alpha _V + \gamma _V ) P_V . \end{aligned}$$

The parameters are as defined in Table 1 and, where applicable, the subscripts denote the strain corresponding to the parameter. The pair equations are in Appendix B.2 defined by Eq. B4-B13. Once again, the system is closed using a pair approximation as outlined in Section “Model” (Matsuda et al. 1992; Satō et al. 1994).

Initially, we assume the lattice is comprised of sites in states 0, *S* or *I*, and that the system is at an endemic steady state expressed by $P_i^*$, $P_{ij}^*$, $q_{i/j}^*$ with $P^*_V = P^*_{iV} =q^*_{i/V}=q^*_{V/i}= 0 \quad \forall i\in \{0,S,I,V\}$. At time $t_M$, a mutant is introduced into the population through one of three event types as defined in Table 2. Event type *S* is when during horizontal transmission the pathogen mutates. Event type *I* occurs when the pathogen mutates within an infected host. Biologically, we consider this type to be most likely. And for event type 0, a host with the mutant strain migrates into the population. We assume that the local environment of the mutant is the same as the site-type from which it mutated.

**Table 2:** The three different event types for which a mutant may be introduced into the population. Note that by the pair approximation, we have that $q_{i/V} = q_{i/VI} = q_{i/SI} = q_{i/S}$

Event Type	Process	Local environment assumption $\forall i \in \{0,S,I,V\}$
S	*SI → VI*	$q_{i/VI} = q_{i/SI}$
I	*I → V*	$q_{i/V} = q_{i/I}$
0	*0 → V*	$q_{i/V} = q_{i/0}$

Following the mutant event, the system is defined by $P_i^{(M)}$, $P_{ik}^{(M)} \quad \forall i,k\in \{0,S,I,V\}$. For example, $P_V (t_M) = P_V^{(M)}$. For event type *j*, the probability densities are expressed as,

22 $$\begin{aligned} P_j^{(M)}&= P_j^* - P_V^{(M)} \end{aligned}$$

23 $$\begin{aligned} P_i^{(M)}&= P_i^* \quad \forall i \in \{0,S,I\} \backslash \{j\} \end{aligned}$$

24 $$\begin{aligned} P_{iV}^{(M)}&= \frac{P^*_{ij}}{P^*_j} P^{(M)}_V \quad \forall i \in \{0,S,I,V\} \end{aligned}$$

25 $$\begin{aligned} P_{ij}^{(M)}&= P_{ij}^* - P_{iV}^{(M)} \quad \forall i \in \{0,S,I\}\backslash \{j\}\end{aligned}$$

26 $$\begin{aligned} P_{jj}^{(M)}&= P_{jj}^* - 2P_{Vj}^{(M)} \end{aligned}$$

27 $$\begin{aligned} P_{kn}^{(M)}&= P_{kn}^* \quad \forall k,n \in \{0,S,I\} \backslash \{j\} \end{aligned}$$

Following a mutation event, the mutant will either invade and replace the resident strain, invade and co-exist with the resident strain or die out. Initially, consider the case when $\beta _I = \beta _V$, $\alpha _V = \alpha _I$ and $\gamma _V = \gamma _I$, such that there is no cost to vertical transmission. For all three event types, the mutant will always invade and either replace or co-exist with the resident strain. For event type *I*, if $\dot{P_V(t_M)} >0$ then the mutant will invade, which is always the case. For event types *S* and 0, the mutant will invade if $\beta _V q_{S/j}^*+bf\rho q_{0/j}^* > \beta _V q_{S/I}^*$ for event type $j \in \{0,S\}$. Through numerically simulating the time evolution of the model for 16525 parameter sets of which 9720 sets resulted in the initial resident strain invading, these inequalities were found to always hold for event types *S* and 0.

Given vertical transmission is expected to always emerge when cost-free, we now consider the potential for invasion when there is an associated cost. We assume that the mutant strain will have reduced horizontal transmission by assuming $\beta _V = \eta \beta _I$ for $\eta \in [0,1)$. We also assume that virulence and recovery are the same for both strains (i.e. $\alpha = \alpha _I = \alpha _V$ and $\gamma = \gamma _I = \gamma _V$).

**Fig. 6:**
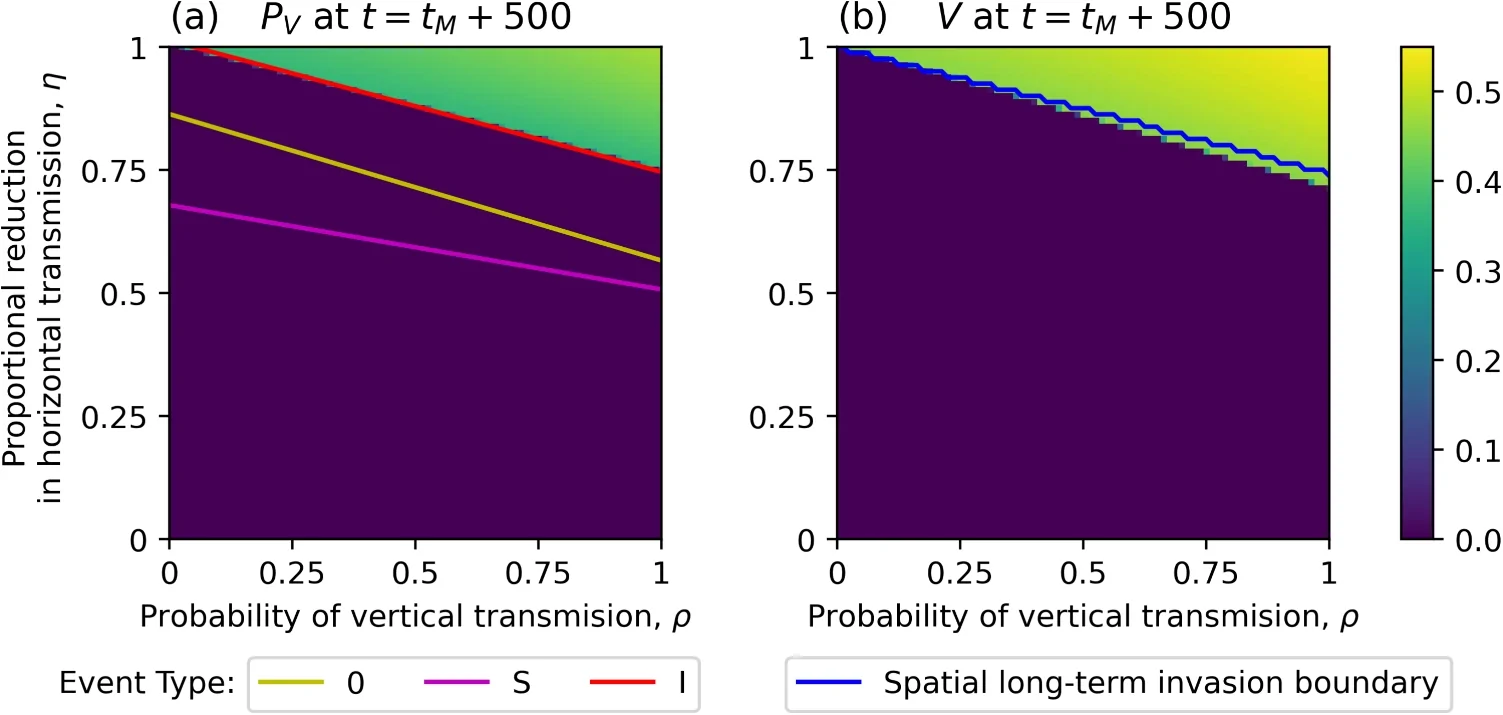
**a** Heatmap showing $P_V$ at $t= t_M + 500$ for the spatial model. The solid lines represent the boundary where $\dot{P_V(t_M)} = 0$. **b** Heatmap showing *V* at $t=t_M+500$ for the mean-field model. The solid line represents the long-term invasion boundary of the spatial model. The fixed parameters for (a) and (b) are $b = 10, f = 0.5, d = 1, \alpha _I = \alpha _V = 0.5, \gamma _I = \gamma _V = 1.5, \beta _I = 8, t_M = 500$ and in (b) *K=1*. The initial conditions for (a) are $(P_0,P_S,P_I,P_V) = (0.1,0.8,0.1,0)$ with the initial pair probabilities as $P_{ij} = P_i P_j$ for $i,j \in \{0,S,I,V\}$ and $P_V^{(M)} = 0.0001$. The initial conditions for (b) are *(S,I,V)=(0.8,0.1,0)* with $V(t_M)=0.0001$

For all three event types the long-term dynamics of the model are identical. Figure 6a shows that for event type *I*, $\dot{P_V(t_M)} > 0$ can be used to predict when the mutant will invade. However, for event types 0 and S, $\dot{P_V(t_M)}>0$ does not correctly predict the long-term dynamics of the model. Due to the mutant appearing in a different environment to other infected hosts, the mutant strain has a brief advantage causing initial growth, even if in the longer-term the mutant is unable to outcompete the resident strain. For example, in event type *S* the mutant is more likely to be surrounded by susceptibles compared to the event type *I* case. A similar logic applies to event type 0 as the mutant is more likely to have neighbouring empty-sites to reproduce into. A similar result was found in the case of a trade-off with virulence (see Appendix C). In both cases, there needs to be a fairly strong cost to vertical transmission for the mutant strain to not invade.

In the mean-field model, defined by Eq. B1-B3 in Appendix B.1, $\dot{P_V(t_M)} > 0$ does accurately predict the long-term dynamics of the model. The mean-field model has a similar invasion threshold when compared to the spatial model, with a difference only occurring for higher *ρ *. Therefore, the mutant is less likely to invade in the spatial model. The final density of the mutant is lower in the spatial model.

## Discussion

We have introduced spatial structure into a host-parasite system with horizontal and vertical transmission. By including spatial structure, we observe the possibility of endemic limit cycles. Furthermore, the amount of spatial structure affects the invasion boundary between the disease-free and endemic states, as well as the importance of vertical transmission for invasion.

One focus of our study has been the conditions that will allow for the initial emergence of vertical transmission. In the absence of cost, we found such a mutation to commonly be a favourable strategy, because the mutant population either co-existed with or displaced the resident strain. Biologically, it is more likely that there is a cost to vertical transmission, and this has been observed as a trade-off with either virulence (Cobos et al. 2019; Stewart et al. 2005) or horizontal transmission (Ebert 2013; Herre 1995; Jaenike 2000; Stewart et al. 2005; Turner et al. 1998; Zilio et al. 2018). For a basic linear trade-off, we find that the mutant is still likely to emerge. Notably, however, due to spatial structure, the initial growth of the mutant strain does not always predict the long-term dynamics. If the mutation occurs during horizontal infection, the mutant will have a local environment identical to that of a susceptible host. This gives the mutant an advantage over the resident strain due to the clustering of susceptible hosts in space. Similarly, if the host infected with the mutant strain emerges in an empty site, perhaps via migration, its local environment will match that of empty sites, giving the mutant host ample ability to reproduce and vertically transmit the pathogen. In both cases, having a more advantageous initial local environment may be insufficient for long-term growth of the mutant. When the mutation occurs within an infected host this initial advantage is absent and the initial growth of the mutant accurately predicts the long-term dynamics. Despite these early differences, the long term dynamics are identical for each mutation event, which suggests the way the mutant emerges only influences the initial dynamics of the mutant strain.

In comparing the mean-field model to the spatial, we see that the mutant invasion boundaries differ only when the mutation is for higher levels of vertical transmission. Therefore, when the mutation is for low levels of vertical transmission, if the mutant can invade a mean-field system it can invade the spatial also. When the mutation is for higher levels of vertical transmission, dependent on the cost, a mutant that can persist in the mean-field may not persist in the spatial model. Secondly, the mutant strain has a lower final density in the spatial model, compared to the mean-field. This is expected because disease prevalence is lower in spatial models (Webb et al. 2007a).

We might therefore conclude that a mutant with vertical transmission as an additional transmission mode is likely to invade, which raises the question asked by Ebert (2013) as to why pathogens with multiple transmission modes are not observed more? Lab studies have shown that additional transmission routes are beneficial to pathogen invasion and persistence (Mangin et al. 1995). The lack of examples observed may be due to some unknown cost, trade-off or biological restriction not accounted for in current evolutionary studies. Negative trade-offs between vertical and horizontal transmission have been observed in specific ecological contexts (Herre 1995; Jaenike 2000; Stewart et al. 2005; Turner et al. 1998; Zilio et al. 2018) and explored within mathematical models (Mangin et al. 1995; Bosch et al. 2010). However, there have been cases where the trade-off has been positive (Ebert 2013). Here we considered only the negative trade-off because in populations that are more spatially-structured the trade-offs observed have been negative (Ebert 2013; Herre 1995; Jaenike 2000; Stewart et al. 2005).

Our results suggest that a mutant with any level of vertical transmission can persist in a spatially-structured system, if the mutant strain has a horizontal transmission rate above a certain threshold. Therefore, a strain with low or high vertical transmission could invade. This does not rule out the possibility of the bistability observed in a mean-field model (Bosch et al. 2010) occurring within a spatially-structured model. A natural extension of our analysis here would be to develop a full evolutionary model of vertical transmission in a spatially-structured population. This would allow a more direct comparison to mean-field evolutionary models that include vertical transmission within the trade-off (Bernhauerová and Berec 2015; Lipsitch et al. 1996; Bosch et al. 2010).

In both mean-field (Busenberg et al. 1983; Lipsitch et al. 1995) and spatial models a pathogen cannot invade with vertical transmission alone. Although, in both cases, the presence of vertical transmission reduces the minimum horizontal transmission rate required for invasion. However, this threshold horizontal transmission rate is affected by spatial structure. To investigate this, and other effects of spatial structure, our model includes both local and global processes. Similarly to Webb et al. (2007a), we find that increasing the proportion of local transmission and reproduction increases the horizontal transmission rate required for invasion. Furthermore, we find vertical transmission behaves similarly. While the level of local reproduction is closely tied to vertical transmission, the proportion of local horizontal transmission has a larger impact on the minimum probability of vertical transmission required for pathogen invasion. This is likely because local transmission is closely correlated to horizontal transmission, which is the dominant transmission mode in the early stages of an epidemic. As mentioned above, we found that there are horizontal transmission rates where vertical transmission is not a requirement for parasite invasion, in agreement with the 2-dimensional stochastic model of Schinazi (2000). However, by including local and global transmission we observed that if a pathogen in a mean-field system does not require vertical transmission, this might not necessarily be the case for more localised systems. In contrast to our results, Schinazi (2000) found that for a 1-dimensional lattice, if vertical transmission is below a critical threshold then regardless of the horizontal transmission rate the pathogen will not invade, suggesting that the precise details of the spatial structure may be crucial.

Using the same methodology as Lipsitch et al. (1995), we explore the different roles horizontal and vertical transmission play throughout an epidemic in a spatially-structured system. In both mean-field and spatial contexts, horizontal transmission is dominant in the early stages and vertical transmission is dominant when disease prevalence is high. Compared to the mean-field model, the spatially-structured system has more cases caused by horizontal transmission occurring at the steady state. This is because for a fixed horizontal transmission rate, in a spatially-structured system susceptible host density is higher compared to the mean-field. The swapping of dominant transmission modes between horizontal early on and vertical transmission at the steady state, suggests that different control strategies will be required depending on the epidemic stage (Aliabadi et al. 2011; Cardenas et al. 2023; Idigo et al. 2025; Veronese et al. 2021).

We also observe that increasing the probability of vertical transmission reduces the number of new cases caused by horizontal transmission in the later stages of an epidemic. When vertical transmission is absent, infected reproduction results in infected hosts gaining susceptible neighbours. However, when vertical transmission is present, infected reproduction may result in more infected neighbours thus reducing the availability of susceptible hosts. Higher horizontal transmission rates also result in a decreased availability of susceptible hosts, which in turn increases the proportion of vertical transmission at the steady state. Therefore, if disease prevalence is extremely high and susceptible host density is low, then vertical transmission is likely to be the primary driver behind new cases. In their mean-field model, Lipsitch et al. (1995) present a case where only vertical transmission is contributing to new cases at the steady state due to there being no susceptible hosts remaining, and therefore total infection occurring. When local processes are included, the same parameter values result in a steady state where horizontal transmission is still contributing to new cases. Spatial structure is known to result in slower rates of disease spread (Keeling 1999), in turn leading to lower infected and higher susceptible densities compared to the mean-field (Webb et al. 2007a), leading to the potential for ongoing horizontal transmission. Total infection has been observed within a spatial model (Silva et al. 2017), however, we do not observe it in our model because imperfect vertical transmission and low levels of recovery are sufficient to maintain a susceptible host population even when horizontal transmission is high.

As in previous studies of spatially-structured host-parasite dynamics (Webb et al. 2007a, b), we find that limit cycles occur, although only under specific parameter conditions. For example, when a high proportion of transmission and reproduction are local, and when infecteds’ fecundity is low. In particular, we observe that local reproduction has a larger effect on limit cycles compared to local horizontal transmission (Webb et al. 2007a). We have identified that vertical transmission can play a key role in the occurrence of limit cycles, where higher levels of vertical transmission are more likely to lead to cycles. Reducing the probability of vertical transmission results in more susceptible offspring from infected hosts, which Webb et al. (2007b) found to stabilise limit cycles. However, to observe the effects of vertical transmission, our model needs to include an adequate level of infected reproduction. Therefore, limit cycles are less likely in our model compared to Webb et al. (2007b).

For both mean-field density- and frequency-dependent horizontal transmission, vertical transmission allows the pathogen to invade over a broader range of ecological conditions and can result in total infection (Altizer and Augustine 1997; Lipsitch et al. 1995). We have only investigated the case of density-dependent horizontal transmission, so it is not known whether in the spatial case the results will be consistent between density- and frequency-dependant horizontal transmission. Alternative birth functions and spatial structure may also give rise to different behaviours.

A potential extension of our model would be to include spatial heterogeneity. For perfect vertical transmission, Su et al. (2019) found habitat loss to increase the importance of vertical transmission in disease prevalence. This is likely due to habitat loss decreasing the availability of susceptible hosts. If imperfect vertical transmission were included, then we would expect that the availability of susceptible hosts would increase and that the pathogen could persist at higher levels of habitat loss.

In summary, our findings show that vertical transmission’s role in pathogen invasion and the occurrence of limit cycles is dependent on the amount of spatial structure within a system. As in the mean-field case, for a spatially-structured system vertical transmission may also be the dominant cause of new cases when disease prevalence is high. Our results also suggest that evolving vertical transmission routes may be a favoured evolutionary strategy in a highly spatially-structured system, which could be investigated further via an evolutionary spatial model.

## Supplementary Information

Below is the link to the electronic supplementary material.

Supplementary file 1 (PDF 504 KB)

## Data Availability

No datasets were generated or analysed during the current study.
