# Supplementary material for: The effects of spatial structure on a host-parasite model with horizontal and vertical transmission
Source: Theor Ecol. 2026 Oct 10;19(1):17. doi: 10.1007/s12080-026-00645-y (PMC13653842; doi:10.1007/s12080-026-00645-y)
Supplement: Supplementary file 1 [file 12080_2026_645_MOESM1_ESM.pdf]

Appendix A Limit Cycles

First we look at how fecundity and vertical transmission affect the occurrence of limit cycles. Fig. A1 shows that limit cycles only occur for very small values of f. When vertical transmission is perfect ($\rho = 1$), limit cycles occur for higher values of f compared to when it is imperfect. This suggests that infected hosts giving birth to susceptible hosts has a stabilising effect on limit cycles. This is consistent with results for when there is no vertical transmission (Webb et al. 2007). To further investigate the role of fecundity, we ran the model for $100,000$ different parameter sets. For $f \in [0, 0.05)$, the maximum value for which limit cycles occurred was 0.028. Cycles occurred for the full range of all other parameter values tested. These were $\beta, b \in [50, 250), \alpha \in [0.0001, 0.1), \gamma \in [0.0001, 0.001), \rho \in [0.75, 1)$ and $L_R, L_T \in [0.9, 1)$. Fig. A2 shows how β and b effect the amplitude of limit cycles.

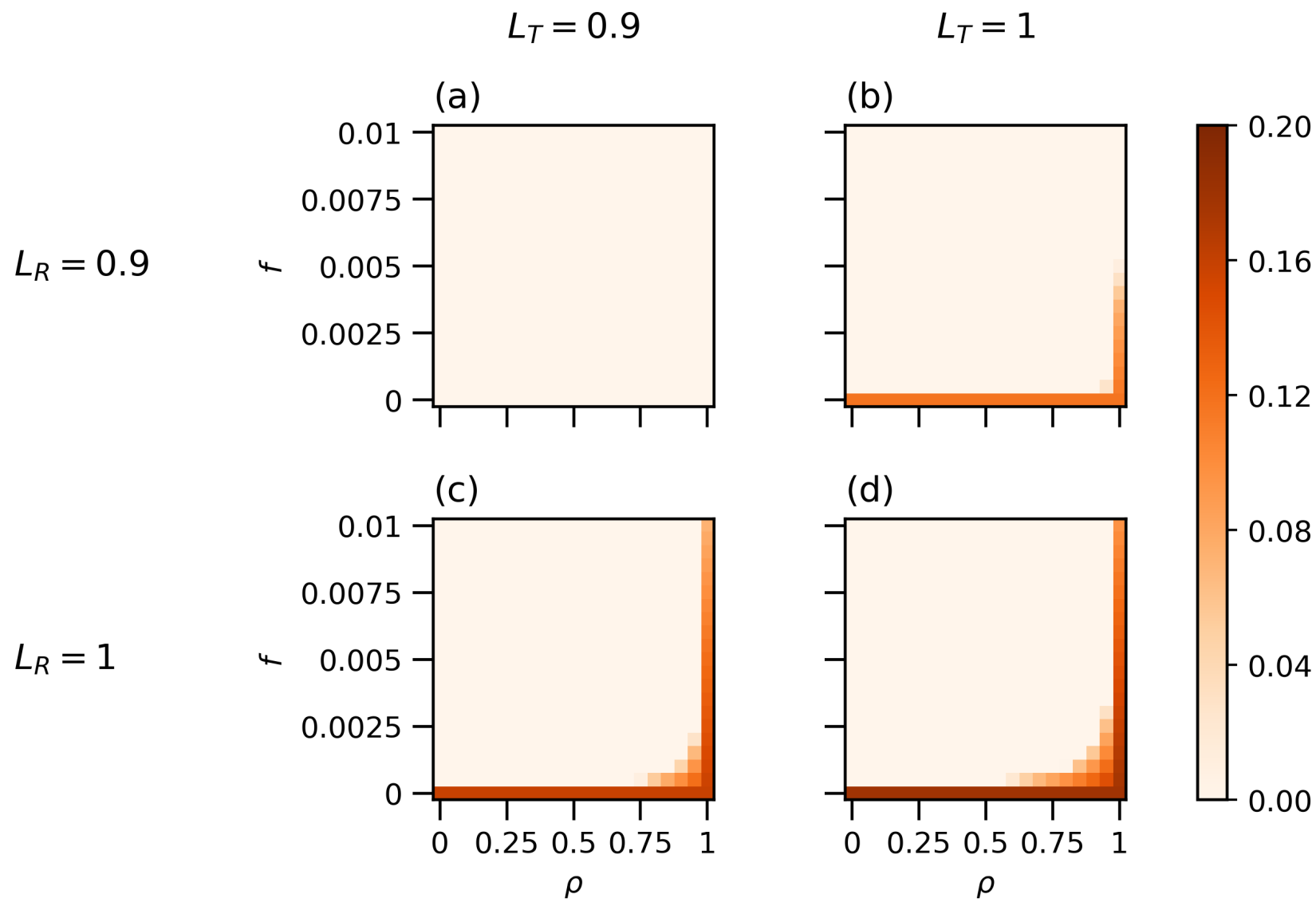

Fig. A1 Heatmaps showing the amplitude of limit cycles. The fixed parameters are $b = 100$, $d = 1, \alpha = 0.05, \gamma = 0.0005$ and $\beta = 100$. The initial conditions are the same as Fig. 1

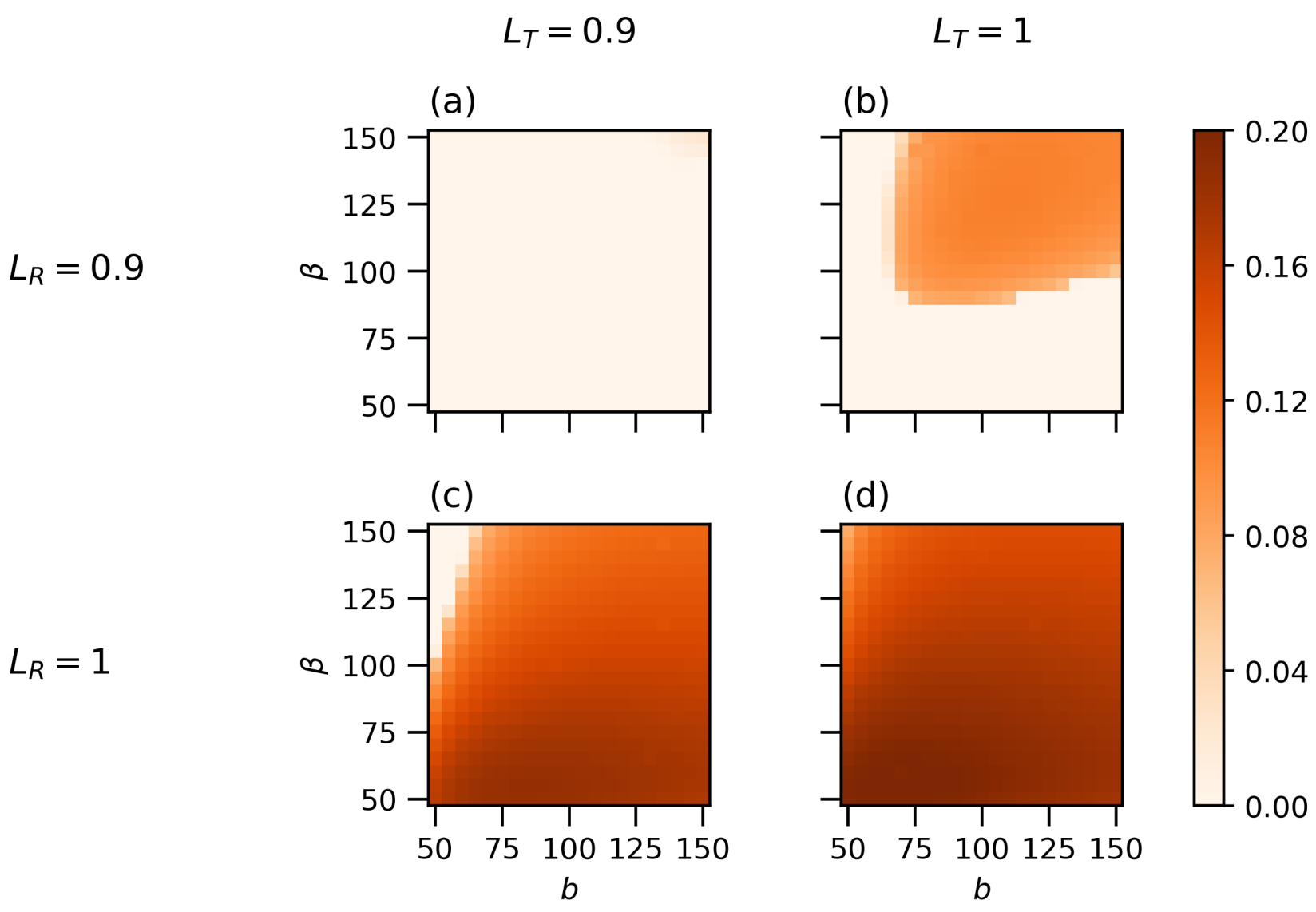

Fig. A2 Heatmaps showing the amplitude of limit cycles. The fixed parameters are $f = 1e-5, d = 1, \alpha = 0.05, \gamma = 0.0005$ and $\rho = 0.75$. The initial conditions are the same as Fig. 1

Appendix B Additional Equations for the Mutant Model

B.1 Mean-field Equations

The mean-field version of the spatial model described in Subsection 3.2 is defined by the equations

$$\dot{S} = b(1 - N/K)(S + fI + f(1-\rho)V) - \beta_I SI - \beta_V SV + \gamma_I I + \gamma_V V - dS \tag{B1}$$
$$\dot{I} = \beta_I SI - (d + \alpha_I + \gamma_I)I \tag{B2}$$
$$\dot{V} = b(1 - N/K)f\rho V + \beta_V SV - (d + \alpha_V + \gamma_V)V. \tag{B3}$$

The parameters are as defined in Subsection 2.1 and, where applicable, the subscripts denote the strain corresponding to the parameter.

B.2 Pair Equations

The corresponding pair equations for the system defined by Eq. 18-21 are

$$\begin{aligned}\dot{P_{00}} = {} & 2dP_{S0} + 2(d + \alpha_I)P_{I0} + 2(d + \alpha_V)P_{V0} \\ & - 2(3/4)(bq_{S/00}P_{00} + bfq_{V/00}P_{00})\end{aligned} \tag{B4}$$

$$\dot{P_{S0}} = dP_{SS} + (d+\alpha_I)P_{SI} + (d+\alpha_V)P_{SV} + (3/4)b(q_{S/00} + f(1-\rho)q_{V/00})P_{00} + \gamma_I P_{I0} + \gamma_V P_{V0} - b(1/4 + (3/4)q_{S/0S} + (3/4)fq_{V/0S})P_{S0} - (3/4)(\beta_I q_{I/S0} + \beta_V q_{V/S0})P_{S0} - dP_{S0} \tag{B5}$$

$$\dot{P_{I0}} = dP_{SI} + (d+\alpha_I)P_{II} + (d+\alpha_V)P_{IV} + (3/4)\beta_I q_{I/S0}P_{S0} - (3/4)b(q_{S/0I} + fq_{V/0I})P_{I0} - (d+\alpha_I+\gamma_I)P_{I0} \tag{B6}$$

$$\dot{P_{V0}} = (3/4)bf\rho q_{V/00}P_{00} + (3/4)\beta_V q_{V/S0}P_{S0} + dP_{SV} + (d+\alpha_I)P_{IV} + (d+\alpha_V)P_{VV} - (d+\alpha_V+\gamma_V)P_{V0} - b((3/4)q_{S/0V} + f(1/4 + (3/4)q_{V/0V}))P_{V0} \tag{B7}$$

$$\dot{P_{SI}} = (3/4)b(q_{S/0I} + f(1-\rho)q_{V/0I})P_{I0} + \gamma_I P_{II} + \gamma_V P_{IV} + (3/4)\beta_I q_{I/SS}P_{SS} - dP_{SI} - \beta_I(1/4 + (3/4)q_{I/SI})P_{SI} - (3/4)\beta_V q_{V/SI}P_{SI} - (d+\alpha_I+\gamma_I)P_{SI} \tag{B8}$$

$$\dot{P_{SV}} = b((3/4)q_{S/0V} + f(1-\rho)(1/4 + (3/4)q_{V/0V}))P_{V0} + \gamma_I P_{IV} + \gamma_V P_{VV} + (3/4)bf\rho q_{V/0S}P_{S0} + (3/4)\beta_V q_{V/SS}P_{SS} - dP_{SV} - (3/4)\beta_I q_{I/SV}P_{SV} - \beta_V(1/4 + (3/4)q_{V/SV})P_{SV} - (d+\alpha_V+\gamma_V)P_{SV} \tag{B9}$$

$$\dot{P_{IV}} = (3/4)\beta_I q_{I/SV}P_{SV} + (3/4)bf\rho q_{V/0I}P_{I0} + (3/4)\beta_V q_{V/SI}P_{SI} - (d+\alpha_I+\gamma_I)P_{IV} - (d+\alpha_V+\gamma_V)P_{IV} \tag{B10}$$

$$\dot{P_{SS}} = 2b(1/4 + (3/4)q_{S/0S} + (3/4)f(1-\rho)q_{V/0S})P_{S0} + 2\gamma_I P_{SI} + 2\gamma_V P_{SV} - 2dP_{SS} - 2(3/4)(\beta_I q_{I/SS} + \beta_V q_{V/SS})P_{SS} \tag{B11}$$

$$\dot{P_{II}} = 2\beta_I(1/4 + (3/4)q_{I/SI})P_{SI} - 2(d+\alpha_I+\gamma_I)P_{II} \tag{B12}$$

$$\dot{P_{VV}} = 2bf\rho(1/4 + (3/4)q_{V/0V})P_{V0} + 2\beta_V(1/4 + (3/4)q_{V/SV})P_{SV} - 2(d+\alpha_V+\gamma_V)P_{VV}. \tag{B13}$$

Appendix C Virulence Trade-Off

Here we consider the case where there is a trade-off between vertical transmission and virulence. We assume that the mutant strain has increased virulence, such that $\alpha_V = \varphi\alpha_I$ where $\varphi > 1$. As in the horizontal transmission trade-off case, for event types S and 0 the condition on $\dot{P_V}(t_M) > 0$ does not accurately predict the long term dynamics of the mutant. Whereas for event type I, $\dot{P_V}(t_M) > 0$ does predict the long term dynamics. Once again, we attribute this difference to the initial advantage in the local environment of the mutant compared to the resident strain. As in the horizontal transmission trade-off case, the long-term dynamics are the same for all three event types.

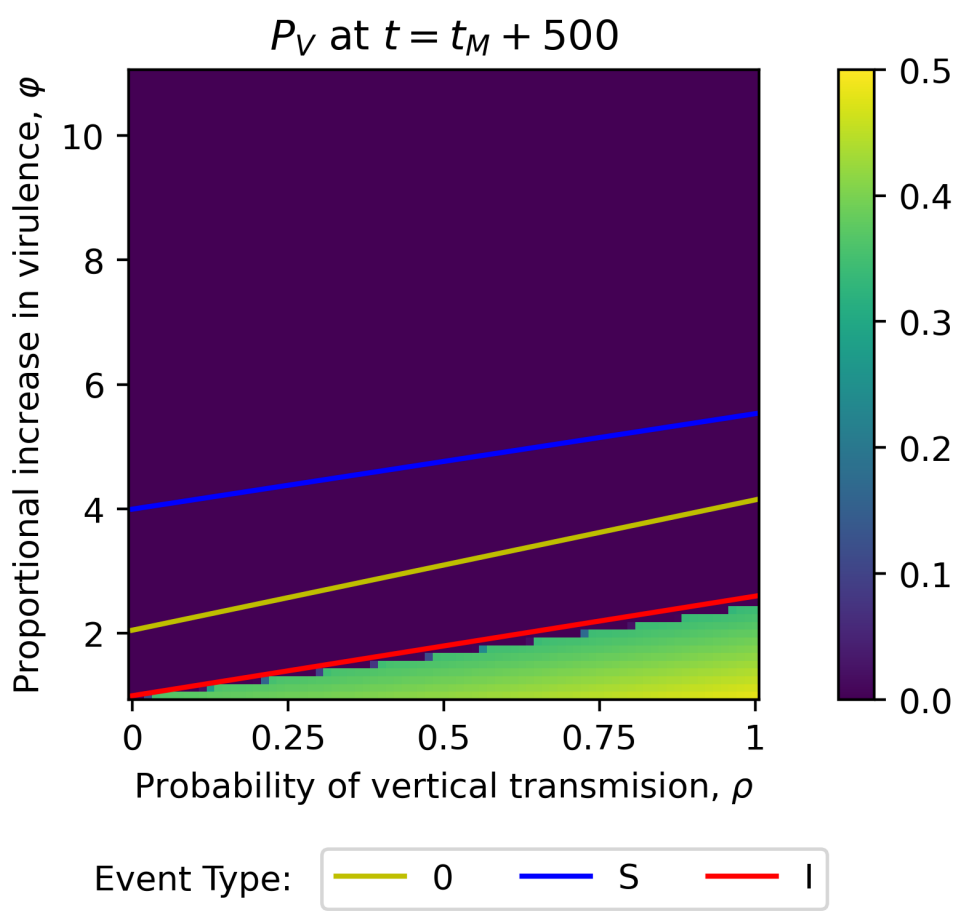

Fig. C3 Heatmap showing P_V at $t = t_M + 500$ for the spatial model. The solid lines represent the boundary where $\dot{P}_V(t_M) = 0$. The fixed parameters are $b = 10, f = 0.5, d = 1, \alpha_I = 0.5, \gamma_I = \gamma_V = 1.5, \beta_I = \beta_V = 8, t_M = 500$. The initial conditions are the same as Fig. 6a

References

Webb, S.D., Keeling, M.J., Boots, M.: Spatially extended host–parasite interactions: the role of recovery and immunity. Theoretical Population Biology **71**, 251–266 (2007) https://doi.org/10.1016/j.tpb.2006.07.010
